# Oral Chinese herbal medicine in reducing the recurrence of colorectal adenoma after polypectomy: A protocol for the systematic review and meta-analysis

**DOI:** 10.1371/journal.pone.0293244

**Published:** 2023-10-20

**Authors:** Yi Cheng, Yuan Ming Di, Anthony Lin Zhang, Beiping Zhang, Charlie Changli Xue

**Affiliations:** 1 China-Australia International Research Centre for Chinese Medicine, School of Health and Biomedical Sciences, STEM College, RMIT University, Melbourne, Victoria, Australia; 2 Department of Gastroenterology, Guangdong Provincial Hospital of Chinese Medicine, The Second Affiliated Hospital of Guangzhou University of Chinese Medicine, Guangdong Provincial Academy of Chinese Medical Sciences, Guangzhou, Guangdong Province, China; Xiamen University - Malaysia Campus: Xiamen University - Malaysia, MALAYSIA

## Abstract

**Background:**

Colorectal adenoma (CRA) is a significant precancerous lesion of sporadic colorectal cancer (CRC). CRA is likely to recur after polypectomy, increasing the risk of CRC. Chinese herbal medicine (CHM) has been used to reduce CRA recurrence. This review aims to evaluate the effectiveness and safety of oral CHM in reducing CRA recurrence compared to other treatments (placebo, routine care, no treatment, and conventional medicine).

**Methods:**

We will search for randomised controlled trials (RCTs) from nine major biomedical databases in English and Chinese from their inception to July 2023. The RCTs that investigate the effects of oral CHM in reducing CRA recurrence compared to other treatments will be involved. We will exclude trials using CHM extract or external application of CHM, cohort study and cross-section study. The Cochrane Risk of Bias Tool version 2 will be used to assess the quality of included studies. Data will be analysed using Review Manager software 5.4 and STATA. The random effect model will be used. The heterogeneity of intervention effects will be tested by Chi^2^ (Cochrane Q) and I^2^ statistics. Funnel plots will assess publication bias if more than ten studies are included. Subgroup and sensitivity analysis will be conducted when possible.

**Discussion:**

This review will discuss the effectiveness and safety of oral CHM in reducing CRA recurrence. It will show the critical information for clinicians in the decision-making process and countries to develop clinical guidelines on CRA management.

**Systematic review registration** PROSPERO CRD42023324197.

## Introduction

Colorectal adenoma (CRA) is a type of colorectal polyp [1]. It is a precancerous lesion that may develop into colorectal cancer (CRC) over time if not managed promptly [2]. There are two types of CRC, sporadic and genetic [2]. About 85%-90% of sporadic CRC is developed from CRA [3]. The incidence rate of CRA among the population >60 years of age is approximately 40%, as shown by a multicentre study in the United States (US) [4]. Another study in the US showed CRA detection rates are between 7.4% to 52.5% in a population of 314,872 [5].

According to the Paris classification, CRA is classified into polypoid and non-polypoid types [6]. To further classify, the polypoid type includes the pedunculated and sessile appearance. Advanced CRA is defined as the adenomatous lesions which meet the criteria: size ≥10 mm, diagnosed with tubulovillous or villous histology, or with high-grade dysplasia [7]. People with advanced adenoma suffer an increased risk of CRC compared to those with non-advanced adenoma [8] and are recommended for short follow-up surveillance of colonoscopy [7]. A study reports that people with 1–2 small adenomas present a lower risk of subsequent CRC than the general population [9]. Those with one or two small, tubular adenomas suffer the average risk of advanced neoplasia [10].

To manage CRA, polypectomy is recommended as the first choice by European and US guidelines [11, 12]. However, the recurrence of CRA after polypectomy is common. A multi-centre study showed that the 1-year recurrence rate of CRA is as high as 59.46% after polypectomy [13]. A large-scale trial (709 participants) in England tested the chemoprevention efficacy of conventional medications and showed that 61% of those who received placebo treatment detected new adenomas [14]. Currently, conventional medications, including calcium, vitamin D, Cox-2 inhibitor, and aspirin, have uncertain efficacy and side effects in reducing CRA recurrence [15–17]. Aspirin is one of the well-researched medications for CRA recurrence. It causes anticoagulation in people with cardiovascular disease [14] and increases the risk of gastrointestinal bleeding for those at an old age [18]. Therefore, it is not recommended for daily use [19].

Chinese herbal medicine (CHM) has been shown to reduce the recurrence of colorectal polyps [20, 21] and even relieve related symptoms, including abdominal distension, abdominal pain, and hematochezia [21]. However, available evidence from meta-analyses is mainly about colorectal polyps, not adenomas [20, 21]. Lin *et al*. summarised that CHM is effective on CRA recurrence in 6-month (Odd ratio (OR) 3.29, 95% confidence interval (CI) 2.30 to 4.69, *P<* 0.0001) and in 1-year (OR 4.21, 95% CI 2.88 to 6.16, *P<* 0.0001) [22]. However, the definition of primary outcome (recurrence rate) in Lin’s study was unclear whether related to CRA. It was the proportion of recurrent adenomas or polyps. The comparison group was limited in treatment options, including fasting and energy support, excluding placebo and other conventional medicine. Additionally, these literature reviews of CHM for colorectal polyps and adenomas were up until 2018 and assessed by version 1 of the Cochrane Risk of Bias tool. In recent years, more rigorous randomised controlled trials (RCTs) of CHM and CRA recurrence have been published, and Cochrane Risk of Bias tool version 2 (RoB 2) has been developed.

Therefore, it is necessary to conduct a systematic review and meta-analysis to update the best available clinical evidence on the effectiveness and safety of oral CHM in preventing CRA recurrence. This paper reports the protocol of a systematic review in evaluating the current evidence on the effectiveness and safety of oral CHM in preventing CRA recurrence compared to other treatments.

## Materials and methods

### Study registration

This review protocol has been registered on PROSPERO (CRD42023324197) and developed by following the Preferred Reporting Items for Systematic Review and Meta-Analysis Protocols (PRISMA-P) [23]. We will update the latest version of this study protocol on the PROSPERO registry if there are any revisions. The conduction of this systematic review will refer to the Preferred Reporting Items for Systematic Reviews and Meta-Analyses (PRISMA) statement checklist [24] and Cochrane Handbook for Systematic Reviews of Interventions [25].

### Inclusion and exclusion criteria

#### Type of studies

We will include individual RCT, which compared the effectiveness and safety of oral CHM and other treatments (placebo, routine care, no treatment, and conventional treatment) for CRA recurrence. The included studies can be published in English or Chinese. Cohort study and cross-section study will be excluded.

#### Type of participants

Participants aged 18 years or older, diagnosed as CRA, evaluated by pathology results showing the tubular, villous, or combination of them (tubulovillous) [26], and have undergone the polypectomy to remove polyps completely will be included. There are no restrictions for gender, race, and nationality.

#### Type of interventions and comparators

Participants with CRA after polypectomy were treated with oral CHM alone or as an add-on therapy to other treatments. We will exclude trials using CHM extract or external application of CHM. CRA participants in the control group can be treated with a placebo, routine care, no treatment, or conventional medicine alone or the combination of the above treatment.

#### Type of outcomes

*Primary outcomes*. The primary outcome is the recurrence rate of CRA. The recurrence rate of CRA is defined as the proportion of participants in each group with CRA detected during the follow-up colonoscopy [27]. The adenoma recurrence can be evaluated at any time, encompassing 6 months, 12 months, or longer than 12 months after the previous colonoscopy.

Secondary outcomes.

1. Number of participants with one to two adenomatous polyps.
2. Number of participants with more than two (not inclusive of two) adenomatous polyps.
3. Number of participants with at least one adenomatous polyp that is 1 cm or greater.
4. Number of participants with at least one advanced adenomatous polyp.
5. Number of participants with a new diagnosis of CRC confirmed by pathological results.
6. Adverse events.

Secondary outcomes 1–5 will be diagnosed by pathological assessment and colonoscopy. The adenomas recurrence number, size and colorectal cancer incidence can be evaluated at any time, usually at 6 months, 12 months, or longer than 12 months after the previous colonoscopy.

### Search methods for identification of studies

#### Data sources

Published studies will be searched. English and Chinese electronic databases for published studies will include PubMed, Cochrane Library, Embase, The Allied and Complementary Medicine Database (AMED), Cumulative Index to Nursing and Allied Health Literature (CINAHL), Chinese Biomedicine Literature Database (CBM), China National Knowledge Infrastructure (CNKI), Chongqing Chinese Science and Technology Periodical Database (CQVIP), and Wanfang Database. The search will be from their inception to July 2023.

#### Search strategy

We will combine various medical subject headings (MeSH) terms and keywords for CRA, CHM and RCT. The proposed search strategy for PubMed is presented in Table 1. We will take this strategy as an example to adapt for use in other electronic databases.

**Table 1:** Search strategy for PubMed.

Search #	Search terms
#1	((Intestinal Polyps [MeSH Terms]) OR (Adenomatous Polyp)) OR (((polyp) OR (adenoma)) AND ((((((colorectal) OR (colorectum)) OR (colon)) OR (rectum)) OR (colonic)) OR (rectal)))
#2	Traditional Chinese Medicine OR Chinese Traditional Medicine OR Chinese Herbal Drugs OR Chinese Drugs, Plant OR Medicine, Traditional OR Ethnopharmacology OR Ethnomedicine OR Ethnobotany OR Medicine, Kampo OR Kampo OR TCM OR Medicine, Ayurvedic OR Phytotherapy OR Herbology OR Plants, Medicinal OR Plant Preparation OR Plant Extract OR Plants, Medicine OR Materia Medica OR Single Prescription OR Chinese Medicine Herb OR Herbal Medicine OR Herbs
#3	randomised controlled trial [pt] OR controlled clinical trial [pt] OR randomised [tiab] OR placebo [tiab] OR drug therapy [sh] OR randomly [tiab] OR trial [tiab] OR groups [tiab]
#4	#1 AND #2 AND #3

### Data collection and analysis

#### Study selection

**Fig 1:**
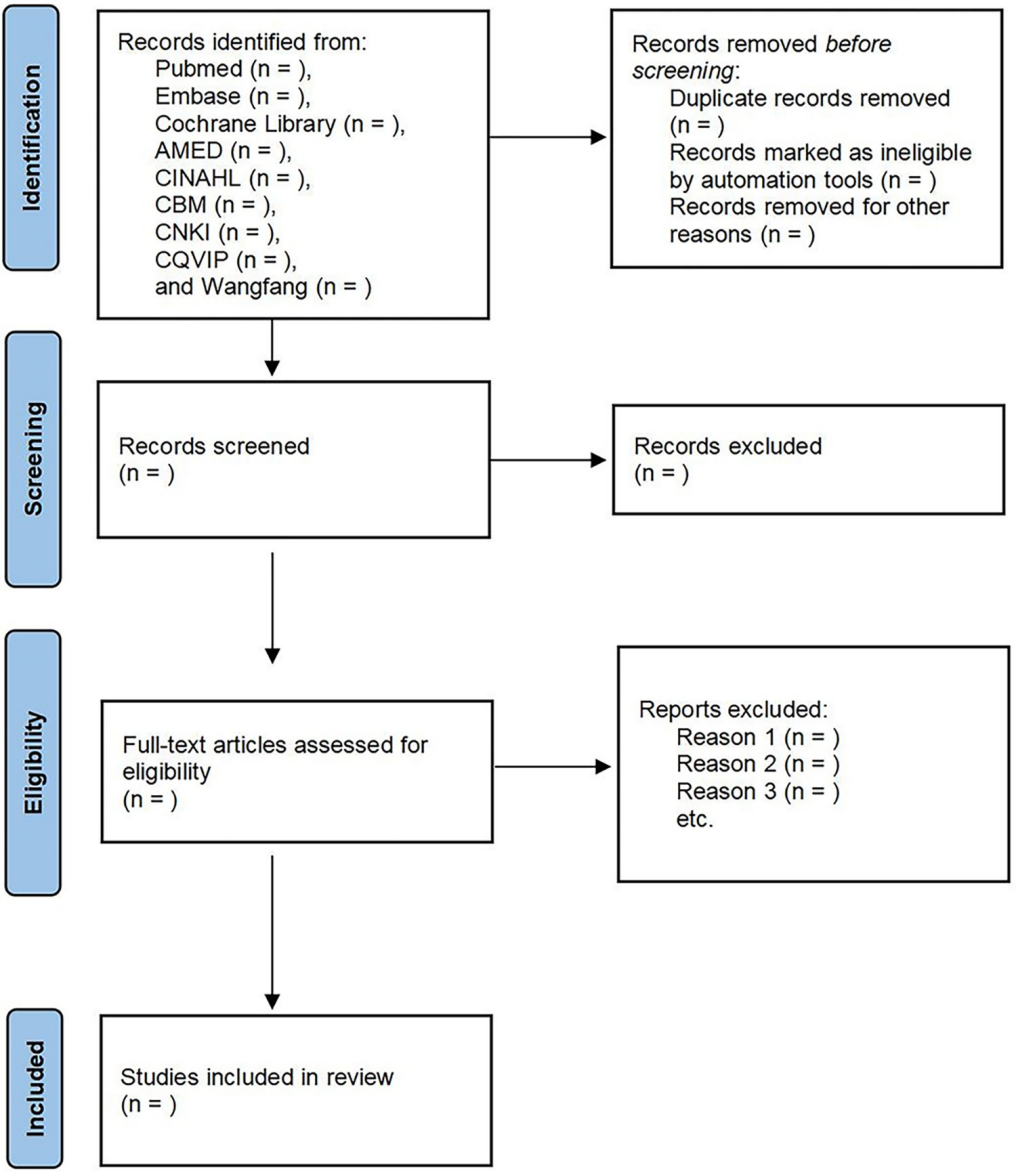
Flow diagram.

Search results from all databases will be downloaded into EndNote 20.0, and duplicate literature will be removed. To select potential studies, we will examine the population, intervention, comparators, and outcome measures. One reviewer (YC) will screen the title and abstract of the search studies for eligibility, which will be double-checked by another reviewer (YMD) independently. The full text of the remaining studies will be read by these two reviewers (YC and YMD) with the inclusion and exclusion criteria. A PRISMA flow diagram will illustrate the details of every step for inclusion and exclusion criteria (see Fig 1) [24].

#### Data extraction

Study data will be extracted in a standardised Excel form. The extracted data will include study characteristics, participants, interventions, and outcomes.

Detailed information is as listed:

- Study characteristics: name of the first author, publication year, country, trial period, study setting, study design, blinding, randomisation, sample size, and groups (treatment and control).
- Participants: average age, age range, number of males and females, diagnosis criteria of CRA, inclusion and exclusion criteria, diagnosis criteria of Chinese medicine syndrome.
- Interventions: oral CHM categories, dosage, formula ingredients, treatment categories of the comparison group, treatment duration, and follow-up period.
- Outcomes: date of repeated colonoscopy, primary and secondary outcomes.

One reviewer (YC) will extract data from the included studies, and another person (YMD) will double-check the extracted data. When necessary, reviewers will contact the original authors to request missing data.

### Quality assessment

Two reviewers (YC and YMD) will independently evaluate the risk of bias for the included studies using the RoB 2 [28]. This tool assesses the risk of bias into five domains around the randomisation process, deviations from intended interventions, missing outcome data, measurement of the outcome and selection of the reported result [28]. Reviewers (YC and YMD) will give domain-level judgement from “low risk”, “some concerns”, or “high risk” and conclude an overall risk assessment. Any disagreement in the quality assessment will be resolved by discussion or consensus with a third reviewer (ALZ).

### Data synthesis and analysis

#### Data analysis

We will give a narrative description of the study results when no sufficient studies are included. Analyses will be performed by the RevMan software (version 5.4) and STATA. Dichotomous outcomes will be measured by risk ratio (RR) with 95% CI for the dichotomous outcomes, including the CRA recurrence rate, number of participants with one to two adenomatous polyps, number of participants with more than two (not inclusive of two) adenomatous polyps, number of participants with at least one adenomatous polyp that is 1 cm or greater, number of participants with at least one advanced adenomatous polyp, number of participants with a new diagnosis of CRC confirmed by pathological results, and adverse events. The numerical variables will be analysed by the difference in means with 95% CI. Data analyses will be performed based on intention-to-treat data. The random-effects model will be used, assuming that the interventions are different. We will evaluate oral CHM as the intervention. Results will be presented in tables and forest plots.

#### Heterogeneity and publication biases assessment

The heterogeneity of intervention effects will be tested by χ^2^ (Cochrane Q) and I^2^ statistics. Heterogeneity comes from the characteristics of participants, interventions, outcomes, the study design, outcome measurement tools, risk of bias and even the intervention effects [25]. We will investigate the heterogeneity and explore the effectiveness of oral CHM by using subgroup analysis or meta-regression. A funnel plot will be used to identify the publication bias if there are ten or more studies.

#### Subgroup analysis

Where possible, we plan to conduct subgroup analyses to explore the source of heterogeneity and the effects of intervention. The subgroup analysis will be based on different Chinese medicine formulae, treatment duration, treatment of comparison group, Chinese medicine syndrome and treatment principles, and participant characteristics.

#### Sensitivity analysis

We plan to perform sensitivity analysis or meta-regression to explore the sources of heterogeneity when necessary. The sensitivity analysis, performed by STATA 17.0, will also explore the robustness of results. We will use the primary outcome for sensitivity analysis by using the leave-one-out method.

#### Quality of evidence

We will use the Grading of Recommendations, Assessment, Development and Evaluations (Cochrane) to classify the overall quality of evidence and strength of recommendations for important outcomes in the analysis [29].

## Discussion

This review will systematically evaluate the current evidence of effectiveness and safety for oral CHM for CRA recurrence, compared to other treatments. Polypectomy of CRA is the primary treatment method [11], but it cannot eliminate the risk of CRC because recurrence of CRA is common [13]. According to clinical guidelines, more attention is required for CRA’s management after polypectomy due to its risk of developing into cancer [7]. Thus, there is a need to seek an effective treatment method to reduce CRA recurrence.

Previous meta-analysis and systematic reviews have reported that CHM is more beneficial than basic treatment for the recurrence of colorectal polyps [20, 21], but not for CRA. Furthermore, more adverse events in the berberine group have been shown in the previous analysis [30], which raises further attention to the safety of CHM. There is a call to summarise the available evidence on the effectiveness and safety of CHM treatment in reducing CRA recurrence.

In this systematic review, we wanted to see if CHM had an effect in risk factors for colorectal cancer, so we included a number of secondary outcomes that can reflect the risk levels for the development of colorectal cancer [7], These include the number of participants with one to two adenomatous polyps, with more than two adenomatous polyps, with at least one adenomatous polyp that is 1 cm or greater, with at least one advanced adenomatous polyp, with a new diagnosis of CRC confirmed by pathological results. Adverse events, another secondary outcome, can reflect the safety of oral CHM for CRA recurrence.

This systematic review has the following limitations. Firstly, the use of CHM is common in China and not as common in Western countries. It will cause the contextual difference in CHM use and lead to an impact on the intervention’s effect. Therefore, this summarised evidence will be more practical in China. Secondly, this systematic review includes RCTs rather than other types of clinical studies to minimise bias. However, the comparison group in some included studies is routine care after polypectomy without any medications related to CRA recurrence. The blinding process is lacking in these studies, this may lead to the overestimation of the effects of the intervention. Lastly, studies with negative results are less likely to be published and summarising the evidence from published studies may cause the overestimation of the effectiveness of oral CHM. Therefore, we will perform a funnel plot to identify the publication bias if there are more than ten (including ten) studies.

This systematic review aims to explore the effectiveness and safety of oral CHM in terms of CRA recurrence. After data synthesis, the findings from this study will be reported in a peer-reviewed journal. We hope this study can summarise the best available evidence on oral CHM and CRA recurrence and provide guidance for clinical research and practice.

## Supporting information

S1 Checklist PRISMA-P 2015 checklist. (DOCX)
